# Prognostic Value of CT-Derived Indicators of Right-Heart Strain and Thrombus Burden for In-Hospital Adverse Events in Acute Pulmonary Embolism

**DOI:** 10.3390/diagnostics16020290

**Published:** 2026-01-16

**Authors:** Corina Cinezan, Camelia Bianca Rus, Alina Cristiana Venter, Angela Cozma

**Affiliations:** 1 Department of Medical Disciplines, Faculty of Medicine and Pharmacy, University of Oradea, 410073 Oradea, Romania; rus.cameliabianca@student.uoradea.ro; 2 Clinical County Emergency Hospital Bihor, 410169 Oradea, Romania; aventer@uoradea.ro; 3 Doctoral School of Biological and Biomedical Sciences, University of Oradea, 410087 Oradea, Romania; 4 Department of Morphological Disciplines, Faculty of Medicine and Pharmacy, University of Oradea, 410073 Oradea, Romania; 5 4th Department of Internal Medicine, Faculty of Medicine, Iuliu Hatieganu University of Medicine and Pharmacy, 400012 Cluj-Napoca, Romania; angela.cozma@umfcluj.ro

**Keywords:** pulmonary embolism, computed tomography pulmonary angiography, right ventricular dysfunction, pulmonary artery obstruction index, inferior vena cava reflux, in-hospital adverse events, prognosis

## Abstract

**Background**: Accurate risk stratification in acute pulmonary embolism (PE) is critical for guiding management. This study assessed the prognostic value of computed tomography (CT) indicators of right-heart strain and thrombus burden for predicting in-hospital adverse events. **Methods**: In this retrospective cohort of 300 patients with CT-confirmed acute PE, the right-to-left ventricular (RV/LV) diameter ratio, Pulmonary Artery Obstruction Index (PAOI), and inferior vena cava (IVC) contrast reflux were measured. The primary endpoint was in-hospital adverse events, including hemodynamic collapse, vasopressor or ventilatory support, rescue reperfusion therapy, or death. Logistic regression and receiver operating characteristic (ROC) analyses were performed. **Results**: Adverse events occurred in 106 patients (35.3%). Compared with stable patients, those with events had higher RV/LV ratios (1.45 vs. 1.03), higher PAOI (38.8 vs. 24.3), and more frequent IVC reflux (74% vs. 7%) (all *p* < 0.001). Independent predictors were RV/LV ratio (aOR 3.22 per 0.1), PAOI (aOR 5.53 per 10 points), and IVC reflux (aOR 428.5; all *p* < 0.001). The model showed excellent discrimination (AUC = 0.96). **Conclusions**: CT-derived indices of right-heart strain and thrombus burden are strong, independent predictors of in-hospital adverse events in acute PE and should be integrated into routine CT-based risk assessment.

## 1. Introduction

PE is a potentially life-threatening condition with a wide spectrum of clinical presentations, ranging from asymptomatic peripheral emboli to sudden cardiovascular collapse [1,2]. Early risk stratification is essential for guiding treatment decisions and determining the need for intensive monitoring or reperfusion therapy. Traditionally, clinical scores and biomarkers such as the Pulmonary Embolism Severity Index (PESI) and cardiac troponins have been used to identify high-risk patients; however, these parameters may not fully capture the hemodynamic consequences of PE [3,4].

Computed tomography pulmonary angiography (CTPA) has become the gold standard for the diagnosis of acute PE, providing not only anatomic confirmation of emboli but also valuable information about right-ventricular function and thrombus burden [5,6]. Several CT-derived indices have been associated with short-term outcomes, particularly the RV/LV diameter ratio, which reflects right-heart strain, and the PAOI, which quantifies clot burden [7,8]. In addition, reflux of contrast medium into the IVC has been described as a qualitative indicator of severe right-ventricular failure [9,10].

Despite the growing use of these imaging biomarkers, their relative and combined prognostic value in predicting in-hospital adverse events remains incompletely defined. Most prior studies have focused on mortality as the primary endpoint, whereas broader adverse clinical events—such as hemodynamic deterioration or need for advanced therapies—are also of major clinical relevance [11,12].

The present study aimed to evaluate the influence of CT-derived parameters, including RV/LV ratio, PAOI, and IVC contrast reflux, on the occurrence of in-hospital adverse events in patients with acute pulmonary embolism. A secondary objective was to assess the diagnostic performance, calibration, and optimal thresholds of these parameters for early risk stratification.

## 2. Materials and Methods

### 2.1. Study Design and Population

This retrospective single-center study included 300 consecutive patients diagnosed with acute PE who underwent CTPA between January 2020 and December 2024. They were admitted and treated in the Cardiology Department of Clinical County Emergency Hospital Bihor, Oradea, Romania.

The diagnosis of PE was confirmed by the presence of a filling defect within the pulmonary arteries on CTPA. Clinical and imaging data were retrieved from institutional electronic medical records and radiology archives.

Patients’ informed consent was waived due to the retrospective nature of the study. At the time of hospital admission, all patients provided general consent allowing the use of their anonymized clinical and imaging data for research purposes. No identifiable personal information was used in the analysis.

### 2.2. Inclusion Criteria

1. Adults (≥18 years) with objectively confirmed acute PE on CTPA.
2. Availability of complete CT imaging data allowing for measurement of RV/LV diameter ratio, PAOI, and IVC contrast reflux.
3. Sufficient clinical data to assess the occurrence of in-hospital adverse events during the index hospitalization.

### 2.3. Exclusion Criteria

1. CTPA of insufficient diagnostic quality due to motion or contrast artifacts precluding quantitative assessment.
2. Chronic or subacute PE (defined by organized thrombus, vessel wall thickening, or mosaic perfusion).
3. Known chronic cardiopulmonary disease (chronic pulmonary hypertension or pre-existing right ventricular dysfunction) that could confound CT-based measurements.
4. Incomplete clinical follow-up or missing in-hospital outcome data.
5. Recurrent admissions for PE during the study period (only the first episode included).

### 2.4. CT Acquisition and Image Analysis

CT pulmonary angiography was performed using a multidetector CT scanner (64 slices, General Electric, GE Health Care, Chicago, IL, USA) according to the institutional PE protocol. Scans were acquired during inspiratory breath-hold following intravenous injection of 60–80 mL of nonionic iodinated contrast at 3–4 mL/s, with bolus tracking in the main pulmonary artery (trigger threshold, 100 HU). Acquisition parameters typically included 120 kVp, automatic tube current modulation, collimation of 0.6–1.0 mm, and 1.0-mm reconstructed slice thickness using a medium-sharp kernel.

**Figure 1:**
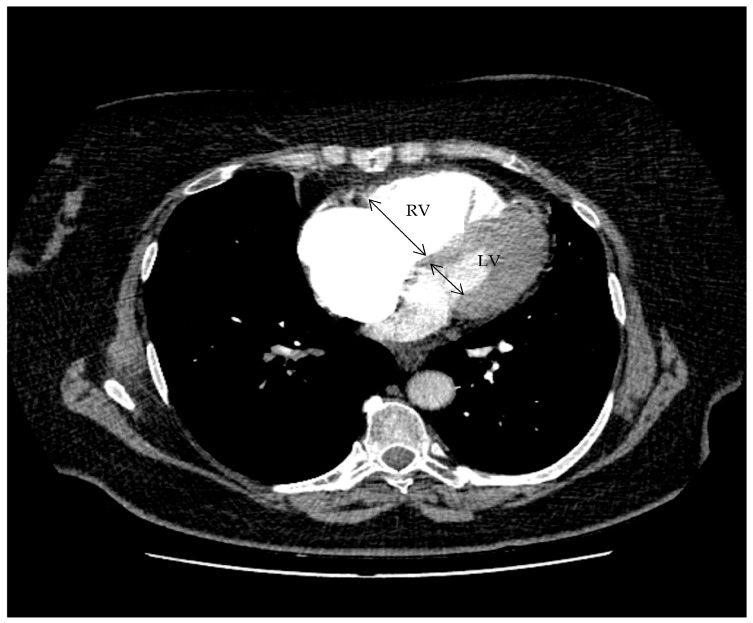
Measurement of the right-to-left ventricular diameter ratio on axial CTPA. Axial contrast-enhanced CT image at the level of the ventricles demonstrating measurement of the maximal transverse diameter of the right ventricle and left ventricle. The RV/LV ratio was calculated as the RV diameter divided by the LV diameter.

The following CT parameters were measured:

• RV/LV diameter ratio: maximum transverse diameter of the right ventricle divided by that of the left ventricle on axial reformats.
• The RV/LV diameter ratio was calculated on axial reformatted images as the ratio between the maximum transverse diameter of the right ventricle and that of the left ventricle, measured at the widest ventricular level. Figure 1 indicates the measurement of the right-to-left ventricular diameter ratio on axial CTPA.
• PAOI: calculated using the Qanadli scoring system (range, 0–40).
• IVC contrast reflux: recorded as present or absent based on retrograde opacification of the inferior vena cava during the pulmonary arterial phase.
• IVC contrast reflux was defined as retrograde opacification of the IVC during the pulmonary arterial phase of CTPA. Reflux into the hepatic veins was not graded separately and was not analyzed as an independent variable.

### 2.5. Explanation of the Qanadli Score (PAOI)

The Qanadli Index, also called the Pulmonary Artery Obstruction Index (PAOI), quantifies clot burden in acute PE on CTPA.

The score works as follows: the pulmonary arterial tree is divided into 10 segmental arteries per lung (20 total). Each segment is scored as1 point if partially occluded and 2 points if completely occluded. The maximum possible score is 40 points (20 segmental arteries × 2 points).

Table 1 presents Qanadli (PAOI) Severity Interpretation.

Notes:

• PAOI = Pulmonary Artery Obstruction Index, calculated as clot involvement relative to segmental arterial branches.
• Higher % indicates greater vascular obstruction and increased right-heart workload.
• Values above 40% correlate with elevated risk of deterioration, shock, or need for reperfusion therapy.
• Thresholds support clinical triage and prognostic stratification.

CT images were evaluated using PACS workstation multiplanar reconstructions.

### 2.6. Clinical Data and Outcomes

Demographic and clinical data, including age, sex, and in-hospital events, were obtained from electronic medical records. This analysis focused exclusively on imaging-derived parameters obtained from CTPA. Clinical, laboratory, and composite prognostic scores such as PESI, troponin, and D-dimer were intentionally excluded to isolate the predictive value of radiologic findings alone. This approach allowed for the evaluation of the independent prognostic potential of CTPA parameters without confounding from clinical variables.

In-hospital adverse events were defined as any of the following occurring during the index hospitalization:

• Hemodynamic collapse or cardiac arrest;
• Requirement for vasopressor support;
• Need for mechanical ventilation;
• Rescue reperfusion therapy (systemic thrombolysis or surgical embolectomy);
• In-hospital death.

### 2.7. Statistical Analysis

Continuous variables are expressed as mean ± standard deviation and compared using Welch’s *t*-test; categorical variables were compared with the chi-square test.

Univariate and multivariable logistic regression models were used to identify predictors of in-hospital adverse events. Variables entered in the multivariable model included age, sex, RV/LV ratio, PAOI, and IVC reflux. Odds ratios (ORs) and 95% confidence intervals (CIs) were calculated.

Because of quasi-separation due to IVC reflux, Firth bias-reduced logistic regression was performed as a sensitivity analysis.

Model discrimination was assessed by the area under the receiver operating characteristic (ROC) curve (AUC), with optimism correction through 200 bootstrap resamples.

Calibration was evaluated with the Brier score and calibration plots using deciles of predicted risk.

Optimal cut-off points for continuous predictors were derived using the Youden index from univariate ROC analysis.

All statistical analyses were conducted in Python 3.11 (pandas, statsmodels, scikit-learn), and a *p*-value < 0.05 was considered statistically significant.

### 2.8. Supplemental Methods

Continuous variables were compared using Welch’s *t*-test and categorical variables using the chi-square test. Univariate and multivariable logistic regression models were fitted to estimate ORs and 95% CIs. Variables included age, sex, RV/LV ratio, PAOI, and IVC reflux. For interpretability, the RV/LV ratio was scaled by 0.1 units and PAOI by 10 points. Firth bias-reduced logistic regression was used to address near-separation caused by IVC reflux. Model discrimination was assessed using the area under the ROC curve (AUC) and bootstrap internal validation (200 samples). Calibration was evaluated using calibration plots and the Brier score. Optimal cut-off points for continuous predictors were determined using the Youden index.

## 3. Results

A total of 300 patients with acute pulmonary embolism were included in the study. In-hospital adverse events occurred in 106 patients (35.3%). These adverse events included hemodynamic collapse, requirement for vasopressor or ventilatory support, rescue thrombolysis or surgical embolectomy, and in-hospital death (Table 2).

Table 3 summarizes the baseline characteristics according to the occurrence of in-hospital adverse events. Patients who experienced adverse events had markedly higher RV/LV ratio and PAOI, and were more likely to present with IVC contrast reflux.

Measurements were independently performed by two experienced thoracic radiologists (each with >8 years of experience), blinded to clinical outcomes. Inter-observer agreement was excellent for RV/LV ratio (intraclass correlation coefficient [ICC] = 0.91) and PAOI (ICC = 0.88), and very good for IVC reflux assessment (Cohen’s κ = 0.82). Discrepancies were resolved by consensus.

In univariate analyses (Table 4), higher RV/LV ratio, greater PAOI, and presence of IVC reflux were significantly associated with in-hospital adverse events. Age and sex were not significant predictors.

In the multivariable logistic regression model adjusting for age, sex, RV/LV ratio, PAOI, and IVC reflux (Table 5), RV/LV ratio (adjusted OR 3.22 per 0.1 increase, 95% CI 2.29–4.52, *p* < 0.001), PAOI (adjusted OR 5.53 per 10-point increase, 95% CI 3.44–8.89, *p* < 0.001), and IVC reflux (adjusted OR 428.45, 95% CI 86.46–2123.33, *p* < 0.001) were independent predictors of in-hospital adverse events. Age and sex were not independently associated with adverse outcomes.

**Figure 2:**
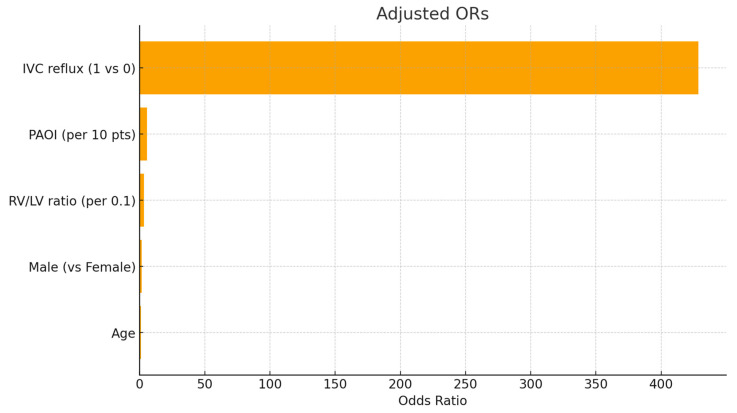
Adjusted odds ratios for CT-derived predictors of in-hospital adverse events. Forest plot showing multivariable-adjusted odds ratios (95% CI) for RV/LV ratio (per 0.1), PAOI (per 10 points), and presence of IVC reflux. All imaging markers were significant independent predictors of adverse clinical events.

Figure 2 depicts the adjusted odds ratios with 95% confidence intervals for the main CT-derived predictors of in-hospital adverse events: RV/LV ratio (per 0.1 increase), PAOI (per 10-point increase), and the presence of IVC reflux. The effect sizes are shown on a logarithmic scale, visually demonstrating that IVC reflux is the strongest independent predictor, followed by PAOI and RV/LV ratio.

Because IVC reflux nearly separated outcomes, Firth bias-reduced logistic regression was used as a sensitivity analysis (Table 6). The associations remained robust, with similar direction and magnitude of effects. The adjusted odds ratio for IVC reflux was reduced to 176.85 (95% CI 38.26–817.53), confirming the stability of the main findings.

Model discrimination was excellent, with an apparent AUC of 0.957.

**Figure 3:**
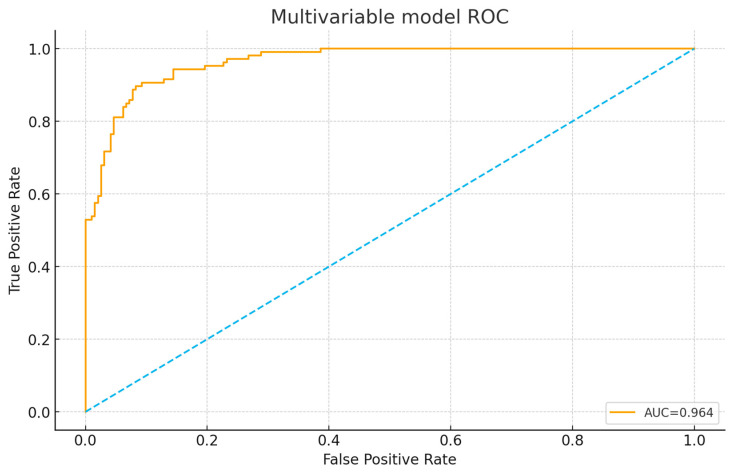
Receiver operating characteristic (ROC) curve for the multivariable prognostic model. The combined CT parameters (RV/LV ratio, PAOI, and IVC reflux) produced excellent discrimination for predicting in-hospital adverse events, with an AUC of 0.964.

Figure 3 presents the receiver operating characteristic (ROC) curve for the multivariable prognostic model combining RV/LV ratio, PAOI, and IVC reflux. The curve demonstrates excellent discrimination, with an area under the curve (AUC) of 0.964.

Bootstrap resampling (200 samples) showed a mean optimism of 0.005, yielding an optimism-corrected AUC of 0.952. The model demonstrated good calibration with a Brier score of 0.086 (Table 7, Figure 3).

Optimal thresholds were derived using the Youden index (Table 8). The optimal RV/LV ratio threshold was 1.22 (sensitivity 0.86, specificity 0.86, AUC 0.935), and the optimal PAOI threshold was 31.6 (sensitivity 0.86, specificity 0.85, AUC 0.928).

## 4. Discussion

This study demonstrates that CT parameters reflecting right ventricular dysfunction and thrombus burden are powerful predictors of in-hospital adverse events in patients with acute PE. Among 300 consecutive patients, 35% experienced adverse events, including hemodynamic collapse, requirement for vasopressor or ventilatory support, rescue thrombolysis, or in-hospital death. Both the RV/LV diameter ratio and PAOI were independently and strongly associated with clinical deterioration. The presence of IVC contrast reflux was an exceptionally strong marker of adverse outcome, reflecting severe right-sided pressure overload.

### 4.1. CT Markers of Right-Heart Strain

The RV/LV diameter ratio on CT is a well-established marker of right-ventricular dysfunction and has repeatedly been linked to short-term mortality and adverse clinical events [5,13,14]. In this study, each 0.1 increase in RV/LV ratio tripled the odds of in-hospital adverse events, confirming the critical role of acute right-ventricular strain in the pathophysiology of PE-related hemodynamic compromise. The optimal cut-off point of 1.22 observed here aligns closely with thresholds reported in prior studies (typically between 1.0 and 1.2), supporting its robustness as a prognostic imaging biomarker.

### 4.2. Thrombus Burden and PAOI

PAOI quantifies the extent of vascular obstruction and indirectly reflects the embolic load [7,15]. A higher PAOI was associated with increased risk of adverse events, with each 10-point increment increasing risk more than fivefold in the adjusted model. This finding reinforces the notion that both embolic load and right-ventricular dysfunction contribute synergistically to early decompensation. The Youden-optimal threshold of approximately 32 provides a practical benchmark for clinical risk stratification.

### 4.3. IVC Reflux as a Sign of Critical Right-Heart Failure

Contrast reflux into the IVC, although less commonly reported in quantitative analyses [11,16], emerged as the single most powerful predictor in this cohort. Nearly all patients with reflux experienced an in-hospital adverse event. This sign likely reflects critically elevated right atrial pressures and near failure of the tricuspid valve to maintain forward flow. Even after bias correction using Firth logistic regression, IVC reflux remained associated with a >100-fold increase in risk, underscoring its importance as an indicator of impending circulatory collapse.

### 4.4. Model Performance and Clinical Implications

The multivariable model demonstrated excellent discrimination (AUC 0.96, optimism-corrected 0.95) and good calibration (Brier score 0.086). The very high AUC observed in this study likely reflects the relatively homogeneous cohort and the strong pathophysiologic link between CT-derived indices and hemodynamic compromise. Although internal bootstrap validation reduced potential overfitting, some degree of model optimism cannot be entirely excluded. External validation in larger, multicenter cohorts will be essential to confirm the generalizability of these findings. These results indicate that routine CT-derived parameters can provide accurate early risk stratification in acute PE without additional imaging or laboratory testing.

The integration of RV/LV ratio, PAOI, and IVC reflux into structured CT reports may assist clinicians in identifying high-risk patients who could benefit from closer monitoring or early reperfusion therapy.

Compared with established clinical risk stratification tools such as the simplified Pulmonary Embolism Severity Index (sPESI) and the European Society of Cardiology (ESC) risk model, our CT-based parameters demonstrated substantially higher discriminative performance (AUC 0.96 vs. typically 0.75–0.85 for clinical models). While sPESI and biomarker-based algorithms rely on physiologic and laboratory data, failing to identify patients with substantial right-ventricular strain or thrombus burden who remain hemodynamically stable at presentation, CT-derived indices offer immediate, objective, and reproducible assessment at the time of diagnosis. Combining these imaging markers with existing clinical scores may further enhance early prognostication [1,17].

Given their quantitative and reproducible nature, these CT parameters could be readily integrated into automated or AI-assisted reporting pipelines. Automated extraction of RV/LV ratio, PAOI, and IVC reflux from CT datasets could facilitate real-time risk scoring, enabling prompt identification of patients requiring intensive management.

### 4.5. Clinical Applicability and Integration with Existing Risk Models

From a practical perspective, the routine reporting of these CT indicators could provide real-time risk information for multidisciplinary teams managing acute PE. Incorporating the RV/LV ratio and PAOI as structured report fields, or extracting them automatically through image-analysis software, would allow radiologists to generate standardized alerts for high-risk cases. This imaging-based stratification could guide early transfer to monitored units, closer hemodynamic observation, or timely initiation of reperfusion therapy.

Future research should focus on prospective, multicenter validation of this CT-based model and explore hybrid strategies that integrate imaging and clinical data. Artificial intelligence-assisted quantification of RV/LV ratio and clot burden could further enhance reproducibility and enable automated clinical decision support. Such integration would help bridge diagnostic imaging and acute care, ensuring rapid and evidence-based management of patients with pulmonary embolism.

### 4.6. Comparison with Previous Studies

Our findings are consistent with a substantial body of evidence demonstrating that CT-derived indicators of right-heart strain and thrombus burden are powerful prognostic markers in acute pulmonary embolism. Numerous prior studies have highlighted the value of the RV/LV ratio in predicting early clinical deterioration. For example, Kang et al. and Becattini et al. identified RV dilation on CTPA as a strong predictor of short-term mortality and adverse outcomes, with cut-offs generally ranging from 1.0 to 1.2 [5,14]. The optimal threshold identified in our cohort (RV/LV ratio 1.22) falls squarely within this established range and showed similarly strong associations with in-hospital events. Our effect size, tripling the risk per 0.1 increase, demonstrates an even more pronounced predictive strength than reported in some earlier cohorts, likely reflecting the high event rate and rigorous measurement protocols in our study.

The prognostic role of clot burden, quantified by the PAOI, has also been well documented. Van der Meer et al. and Qanadli et al. found that higher obstruction indices correlated with both imaging evidence of RV dysfunction and adverse clinical outcomes [7,8].

Our findings align closely with a growing body of literature that underscores the prognostic significance of CT-derived markers of right-heart strain in acute PE. For example, a recent systematic review and meta-analysis performed by Vedovati et al. found that embolic burden on CT, especially central localization, is associated with increased short-term mortality [18].

Several more recent cohort studies, however, lend further support to our results. In a retrospective study of 238 PE patients reported by Chaosuwannakit et al., elevated RV/LV ratio, interventricular septum deviation, increased RV diameter, and IVC contrast reflux on CTPA were all significantly associated with 30-day mortality [19].

Our study reinforces this relationship, showing that each 10-point increase in PAOI was associated with more than a fivefold increase in the odds of in-hospital complications. Notably, the Youden-optimal threshold of 31.6 in our population is lower than the ≥45% cut-off reported by Osman and Abdeldayem for predicting short-term mortality [20], suggesting that PAOI may serve as a sensitive early marker of impending hemodynamic compromise even before mortality risk is evident. Furthermore, parallels with the work of Hajiahmadi et al.—who demonstrated the long-term prognostic implications of PAOI through its correlation with chronic RV remodeling—suggest that clot burden provides prognostic information spanning both acute and chronic phases of PE management [21].

The prognostic significance of inferior vena cava reflux has been emphasized in multiple studies, and our findings corroborate its value as one of the most powerful imaging predictors of clinical deterioration. Aviram et al. first demonstrated that higher IVC reflux grades strongly correlate with increased 30-day mortality [22], and more recent analyses have shown that reflux serves as a highly specific marker of depressed cardiac index and acute right-heart failure [23,24]. In our cohort, the presence of IVC reflux was associated with extremely high odds of in-hospital adverse events, even after applying Firth bias reduction to address quasi-separation. This magnitude of association is consistent with the physiologic interpretation that IVC reflux occurs in settings of severely elevated right atrial pressure and impending circulatory collapse. The consistency of IVC reflux as a predictor across heterogeneous cohorts underscores its potential as a rapid, easily identifiable, qualitative marker in routine CTPA interpretation.

Comparisons with recent integrated imaging–clinical scoring systems also support the relevance of our findings. Yeh et al. demonstrated that incorporating PAOI into a simplified CT-based scoring system significantly improves the prediction of early deterioration compared with the sPESI alone [25]. Our results, notably the high discrimination of the combined CT model (AUC 0.96), are in line with these emerging data supporting the integration of imaging markers into multimodal risk assessment frameworks. In contrast to purely clinical models, which typically achieve AUC values between 0.75 and 0.85, our CT-only model showed substantially superior predictive performance, suggesting that imaging biomarkers capture physiologic perturbations that are not readily apparent from clinical examination or laboratory testing.

Overall, our findings not only corroborate prior findings but also extend them by demonstrating exceptionally strong predictive performance of combined CT-derived metrics in a real-world cohort using in-hospital adverse events—not limited to mortality—as the primary endpoint. The consistency of our findings with the broader literature supports the robustness of these imaging markers and underscores their potential for integration into clinical decision support systems and structured CT reports.

### 4.7. Strengths of the Study

This study has several notable strengths.

First, it integrates comprehensive CT-derived indices—RV/LV ratio, PAOI, and IVC reflux—allowing for simultaneous evaluation of both right-ventricular dysfunction and thrombus burden, which together capture the key hemodynamic determinants of adverse outcomes in acute pulmonary embolism.

Second, the analysis was based on a consecutive real-world cohort of 300 patients, minimizing selection bias and reflecting the routine clinical population encountered in emergency imaging practice.

Third, the use of quantitative and bias-corrected statistical methods, including Firth logistic regression and bootstrap validation, provides robustness against small-sample and separation effects while enhancing the reliability of the findings.

Fourth, the study provides practical clinical thresholds for RV/LV ratio and PAOI, supporting direct translation into risk stratification and decision-making.

Finally, the inclusion of comprehensive model validation—with discrimination, calibration, and internal bootstrap correction—strengthens the reproducibility and generalizability of the proposed CT-based prognostic model.

### 4.8. Limitations

This study has several limitations. First, it was retrospective and single-center, which may limit generalizability. Second, although adverse events were clearly defined, event adjudication was based on chart review rather than prospective monitoring. Third, the exceptionally high odds ratio for IVC reflux suggests quasi-separation and small subgroup size; although Firth bias correction mitigated this effect, future studies with larger cohorts are warranted. Hepatic vein contrast reflux was not evaluated separately, which may have provided additional prognostic stratification in patients with severe right ventricular failure.

Finally, external validation in an independent population is needed to confirm model calibration and transportability.

## 5. Conclusions

CT-derived parameters, specifically the RV/LV ratio, pulmonary artery obstruction index, and inferior vena cava reflux, are powerful, independent predictors of in-hospital adverse events in patients with acute pulmonary embolism. Their combination provides a rapid, objective, and reproducible means of assessing right-heart strain and thrombus burden directly from diagnostic imaging.

Integrating these CT indicators into structured reporting templates or automated analysis pipelines could enable real-time risk stratification at the point of care, improving identification of patients who require intensive monitoring or early reperfusion therapy.

Future multicenter studies and prospective validation are warranted to confirm the generalizability of these findings and to explore hybrid CT-clinical algorithms that combine imaging, hemodynamic, and biomarker data for precision risk assessment in pulmonary embolism.

## Figures and Tables

**Table 1:** Classification scale of pulmonary embolism clot burden measured using the Qanadli PAOI scoring method.

PAOI (%)	Clot Burden	Clinical Meaning
<20%	Low	Small embolic load, limited perfusion impact
20–40%	Moderate	Increasing obstruction, early RV strain risk
>40%	High	Associated with hemodynamic compromise & adverse events
>60–70%	Critical	Often correlates with severe RV failure and instability

**Table 2:** Distribution of in-hospital adverse events in the study population.

Adverse Event	*n* (%)
In-hospital death	30 (10.0)
Vasopressor support	28 (9.3)
Mechanical ventilation	24 (8.0)
Rescue reperfusion therapy	18 (6.0)
Any adverse event	100 (35.3)

**Notes:** Values are presented as absolute numbers and percentages of the total study population (*n* = 300). Patients could experience more than one adverse event during hospitalization; therefore, individual event percentages do not sum to 100%. In-hospital adverse events were defined as hemodynamic collapse or cardiac arrest, requirement for vasopressor support, need for mechanical ventilation, rescue reperfusion therapy (systemic thrombolysis or surgical embolectomy), or in-hospital death. Several patients experienced more than one adverse event, and therefore the individual event counts do not sum to the total number of patients with adverse outcomes.

**Table 3:** Baseline characteristics of in-hospital adverse events.

Variable	No Adverse Events (*n* = 194)	Adverse Events (*n* = 106)	Test	*p* Value
Age (years)	62.3 ± 14.4	62.6 ± 12.5	*t*-test	0.849
RV/LV ratio	1.03 ± 0.16	1.45 ± 0.21	*t*-test	<0.001
PAOI	24.3 ± 9.2	38.8 ± 12.1	*t*-test	<0.001
Male sex, *n* (%)	106 (54.6%)	59 (55.7%)	χ^2^	0.961
IVC reflux, *n* (%)	13 (6.7%)	78 (73.6%)	χ^2^	<0.001

**Notes:** Continuous variables are expressed as mean ± SD. *p*-values are derived from Welch’s *t*-test for continuous variables and chi-square test for categorical variables. RV/LV = right-to-left ventricular diameter ratio; PAOI = Pulmonary Artery Obstruction Index; IVC = inferior vena cava.

**Table 4:** Univariate logistic regression for in-hospital adverse events.

Variable	Unit Change	OR	95% CI (Low)	95% CI (High)	*p* Value
RV/LV ratio (per 0.1)	0.1	1.55	1.35	1.78	<0.001
PAOI (per 10 points)	10	2.04	1.65	2.53	<0.001
Age (per year)	1	1.00	0.98	1.02	0.854
IVC reflux (present vs. absent)	1	10.38	5.83	18.48	<0.001
Male sex (vs. female)	1	1.04	0.65	1.68	0.865

**Notes:** Odds ratios (ORs) and 95% confidence intervals (CIs) were calculated using univariate logistic regression. RV/LV = right-to-left ventricular diameter ratio; PAOI = Pulmonary Artery Obstruction Index; IVC = inferior vena cava. Statistical significance was set at *p* < 0.05.

**Table 5:** Multivariable logistic regression for in-hospital adverse events.

Variable	Unit_Change	OR	CI_Low	CI_High	*p*_Value
Age	1.0	1.007	0.973	1.042	0.684
Male (vs. Female)	1.0	1.712	0.71	4.125	0.231
RV/LV ratio (per 0.1)	0.1	3.216	2.289	4.519	<0.001
PAOI (per 10 pts)	10.0	5.53	3.441	8.887	<0.001
IVC reflux (1 vs. 0)	1.0	428.453	86.455	2123.327	<0.001

**Notes:** Odds ratios (ORs) and 95% confidence intervals (CIs) were derived from a multivariable logistic regression model including age, sex, RV/LV ratio, PAOI, and IVC reflux. Continuous imaging variables were scaled to clinically interpretable units: RV/LV ratio per 0.1-unit increase and PAOI per 10-point increase. The model evaluated the independent contribution of each parameter to the risk of in-hospital adverse events. An OR >1 represents increased odds of adverse events with higher parameter values. Statistical significance was set at *p* < 0.05. RV/LV = right-to-left ventricular diameter ratio; PAOI = Pulmonary Artery Obstruction Index; IVC = inferior vena cava.

**Table 6:** Firth bias-reduced logistic regression.

Variable	Unit_Change	OR	CI_Low	CI_High	*p*_Value
Age (per year)	1.0	1.007	0.974	1.041	0.697
Male (vs. Female)	1.0	1.653	0.703	3.885	0.249
RV/LV ratio (per 0.1)	0.1	3.012	2.188	4.146	0.0
PAOI (per 10 pts)	10.0	5.023	3.214	7.852	0.0
IVC reflux (1 vs. 0)	1.0	307.236	68.643	1375.137	0.0

**Notes:** Firth penalized logistic regression was applied to address near-complete separation caused by the strong association between IVC reflux and adverse outcomes. This method reduces small-sample bias in maximum-likelihood estimates, producing more stable ORs and narrower confidence intervals. Predictors were scaled identically to the main model (RV/LV ratio per 0.1-unit; PAOI per 10-point increase). The direction and magnitude of associations remained consistent with the standard multivariable model, demonstrating the robustness of the findings. RV/LV = right-to-left ventricular diameter ratio; PAOI = Pulmonary Artery Obstruction Index; IVC = inferior vena cava.

**Table 7:** Model performance and calibration.

AUC_Apparent	Optimism_Mean	AUC_Optimism_Corrected	Brier_Score
0.957	0.005	0.952	0.086

**Notes:** Model discrimination was quantified using the apparent area under the receiver operating characteristic curve (AUC). Internal validation was performed using 200 bootstrap resamples to estimate optimism, defined as the average decrease in performance when applying the model to unseen data. The optimism-corrected AUC provides a more conservative estimate of real-world performance. Calibration was evaluated using the Brier score, which measures the mean squared difference between predicted and observed outcomes (lower values indicate better calibration). Together, these metrics demonstrate excellent discrimination and good calibration of the CT-based prediction model.

**Table 8:** Youden-optimal cut-off points for CT predictors.

Marker	Optimal Threshold	Sensitivity	Specificity	AUC	95% CI (AUC)
RV/LV ratio	1.22	0.86	0.86	0.935	0.89–0.97
PAOI	31.6	0.86	0.85	0.928	0.88–0.97

**Notes:** Optimal thresholds were determined by maximizing the Youden index on ROC analysis. AUC = area under the receiver operating characteristic curve; RV/LV = right-to-left ventricular diameter ratio; PAOI = Pulmonary Artery Obstruction Index.

## Data Availability

The data presented in this study are available on request from the corresponding author. The data are not publicly available due to privacy reasons.
